# Frequency and visual outcomes of ocular toxoplasmosis in an adult Brazilian population

**DOI:** 10.1038/s41598-021-83051-0

**Published:** 2021-02-09

**Authors:** Rafael Estevão De Angelis, Maria de Lourdes Veronese Rodrigues, Afonso Dinis Costa Passos, Valdes Roberto Bollela, Milena Simões Freitas e Silva, Bárbara Regina Vieira, Moisés Moura de Lucena, Thais David Moralles, Luciana de Morais Vicente, Gutemberg de Melo Rocha, Rodrigo Jorge, Jayter S. Paula, João M. Furtado

**Affiliations:** 1 Department of Ophthalmology, Otolaryngology and Head and Neck Surgery, Ribeirão Preto Medical School, University of São Paulo, Avenida Bandeirantes, Ribeirão Preto, São Paulo 14049-900 Brazil; 2 Department of Social Medicine, Ribeirão Preto Medical School, University of São Paulo, Ribeirão Preto, São Paulo Brazil; 3 Department of Internal Medicine, Ribeirão Preto Medical School, University of São Paulo, Ribeirão Preto, São Paulo Brazil

**Keywords:** Eye diseases, Infectious diseases

## Abstract

Although ocular toxoplasmosis is a leading cause of posterior uveitis worldwide, there is scarce information about the real-life frequency of ocular lesions, visual outcomes, and risk factors for poor prognosis. We conducted a community-based cross-sectional study with 721 adults living in Cássia dos Coqueiros, Southeast Brazil, consisted of visual acuity measurement, dilated ocular examination, a risk-factor questionnaire, and peripheral blood collection for anti-*T. gondii* serology. Presumed toxoplasmic lesions were recorded on video and analyzed by experienced and masked ophthalmologists. Ocular toxoplasmosis was determined if at least one suspected lesion was appointed by two graders in the presence of positive anti-*T. gondii* serology. Forty-eight eyes (n = 42 participants; 6.7% among those with positive anti-*T. gondii* serology) with ocular toxoplasmosis were found. Most lesions were single (n = 28; 58.3%), peripheral (n = 34; 77.1%) and unilateral (85.7% of participants); no active lesions were found. Older age was associated with lesions larger than one-disc diameter (p = 0.047), and lower social stratum (OR: 2.89; CI 1.2–6.97; p = 0.018) was associated with the presence of toxoplasmic lesions. Although there were no differences in visual acuity between participants and eyes with or without ocular lesions (p > 0.05), unilateral blindness associated with ocular toxoplasmosis was identified in a reduced number of individuals.

## Introduction

Toxoplasmosis is a foodborne disease transmitted by the obligate intracellular parasite *Toxoplasma gondii*, which affects up to one-third of the global population^1^. The disease is transmitted mainly by the ingestion of water, fruits, and vegetables contaminated with oocysts, raw or undercooked meat containing tissue cysts (with bradyzoites), or even vertically^2^. Most affected individuals remain asymptomatic through life, and ocular infection, a common manifestation of the disease, can result in a wide range of manifestations, from no symptoms to blindness^3^.

Ocular toxoplasmosis (OT) is a leading cause of posterior uveitis worldwide^2^ and a leading cause of childhood blindness in Brazil^4^. In Latin America, the disease is known to be not only the most frequent cause of posterior uveitis but the leading cause of uveitis itself, contributing to one out of four uveitis cases seen in tertiary services^5^. Factors such as parasite virulence^6^, host susceptibility^7^, and eating habits are considered to influence the variations seen in seroprevalence and visual outcomes between regions^8^.

Although there are multiple studies in the literature reporting clinical aspects of the disease^9,10^, most of them come from uveitis tertiary centers, where up to one-fourth of affected eyes may present blindness^10^, and concentrate more severe cases than community-based studies. There is scarce information about the real-life frequency of ocular lesions, visual outcomes, and risk factors for poor prognosis of OT^2^. The frequency of OT in community-based studies varies widely, ranging from 1.0% to up to 17.7%, and local risk-factors and different study designs could explain the differences found^11–18^. In this community-based study conducted in a large sample of adults residing in Cássia dos Coqueiros (Southeast Brazil), we investigated the frequency, risk factors, and visual outcomes of OT.

## Results

**Figure 1:**
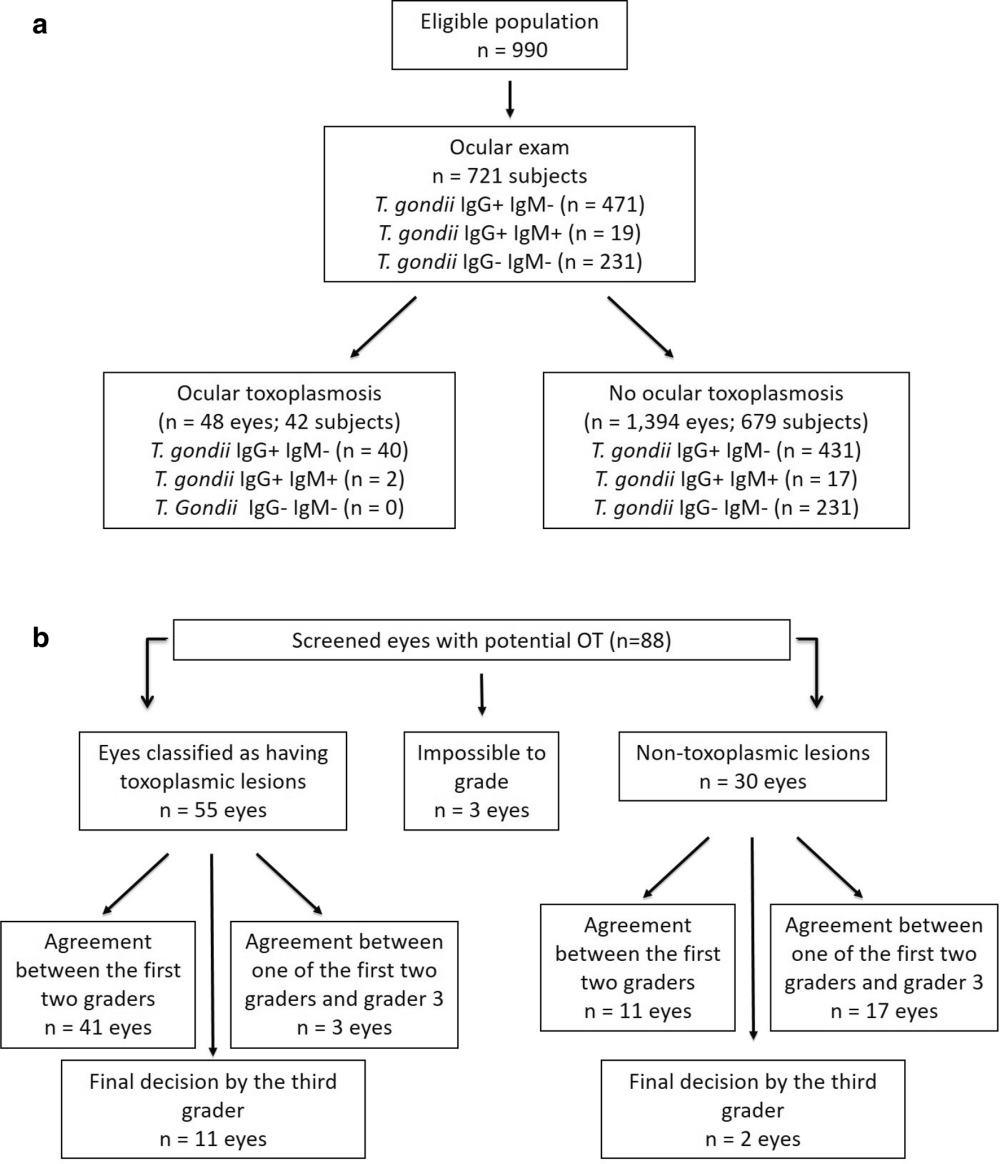
Flowchart of ocular examination: (**a**) Details of the eligible population, number of subjects, serology and ocular lesions found. (**b**) Description of ocular diagnosis and the agreement between examiners.

The number of subjects who accepted the invitation and attended the eye examination was 721 (73.0%), being 450 (62.4%) female. The mean ± standard deviation (SD) of age of the subjects examined was 53.18 ± 15.82 years (range, 22 to 92 years). Most subjects reported living in the urban area (70.3%), had an education level between five and seven years of study (29.1%), belonged to social stratum C1 and C2 (59.6%) (Table 1). Figure 1a describes the relationship between the eligible population, the number of subjects, serology, and ocular lesions found.

**Table 1:** Demographic and socioeconomic characteristics of the population (n = 721 subjects).

Variables	Subjects (%)
**Sex**	
Female	450 (62.4)
Male	271 (37.6)
**Age (years)**	
18–24	15 (2.0)
25–34	89 (12.3)
35–44	116 (16.1)
45–54	167 (23.2)
55–64	159 (22.1)
> 64	175 (24.3)
**Residence**	
Rural	214 (29.7)
Urban	507 (70.3)
**Educational level (years of study)**	
< 4	201 (27.9)
5–7	210 (29.1)
8–10	74 (10.3)
11–15	170 (23.6)
> 15	66 (9.1)
**Social stratum**	
A1	0
A2	8 (1.1)
B1 ou B2	219 (30.4)
C1 or C2	430 (59.6)
D	62 (8.6)
E	2 (0.3)

Social categories classified according to the Brazilian Association of Research Companies (ABEP)^21^, being A1 the highest and E the lowest.

From the subjects screened, eighty-two subjects (88 eyes) had hyperpigmented lesions classified as ‘potential ocular toxoplasmosis’. The graders later examined recorded images of these eyes, and lesions present in 55 eyes (49 subjects) (3.8% of the eyes examined, 6.8% of the subjects) were identified as OT suggestive. The percentage of agreement between the first two graders was 58.23%, with Kappa = 0.37 (p < 0.01) (Fig. 1b). No participant had a lesion suggestive of active toxoplasmosis.

We observed that seven subjects had unilateral lesions considered to be suggestive of ocular toxoplasmosis and negative IgM and IgG *T. gondii* serology, and were invited to repeat the *T. gondii* serology test. However, only four subjects were found and agreed to be submitted to another blood draw (which confirmed the initial serological result); all seven subjects were considered to have no ocular toxoplasmosis since available serology suggested no infection.

Forty-two subjects (5.8%) presented suggestive lesions of OT and positive serology for toxoplasmosis, six with bilateral lesions (48 eyes; 3.3%). Only two of these subjects had *T. gondii* serology IgM and IgG positive, while the remaining 40 subjects had only positivity for the IgG type. Of the 42 subjects who presented ocular toxoplasmosis (n = 48 eyes), six had lesions in both eyes, 19 only in the right eye (OD), and 17 only in the left eye (OS). Twenty-eight eyes (27 subjects) had a single lesion, and 20 eyes (15 subjects) had more than one lesion. Four eyes had only central retinal lesions, 37 eyes had only peripheral lesions, and seven eyes had both central and peripheral lesions (Table 2). Twenty-two subjects (52.4%) had lesions equal to or less than 1 (DD) (ranging from 0.1 to 1 DD) and 20 (47.6%) a lesion larger than 1 DD (ranging from 1.5 to 8 DD).

**Table 2:** Characteristics of subjects and eyes with ocular toxoplasmosis (n = 42 subjects, 48 eyes).

Characteristics	Subjects (%)	OD (%)	OS (%)
**Affected eye**			
OD only	19 (45.2)	19 (40)	–
OS only	17 (40.5)	–	17 (35.4)
Bilateral	6 (14.3)	6 (12.5)	6 (12.5)
Total	42	25	23
**Retinal location**			
Central retina only			
Unilateral	2 (4.8)	1 (2.0)	1 (2.0)
Bilateral	0	0	0
Total	2	1	1
Peripheral retina only			
Unilateral	30 (71.4)	16 (33.3)	14 (29.2)
Bilateral	2 (4.8)	2 (4.2)	2 (4.2)
Total	32	18	16
Central and peripheral retina			
Unilateral	4 (12)	3 (6.2)	2 (4.2)
Bilateral	1 (2.4)	1 (2.1)	1 (2.1)
Total	5	4	3
Peripheral retina OD, central retina OS	2 (4.8)	2 (4.2)	2 (4.2)
Total	2	2	2
Central and peripheral retina OD and peripheral retina OS	1 (2.4)	1 (2.1)	1 (2.1)
Total	1	1	1
**Number of lesions**			
Single lesion	27 (54.8)	16 (33.3)	12 (25)
Multiple lesions	15 (45.2)	9 (18.7)	11 (22.9)
**Size**			
Equal or less than 1 DD	22 (52.4)	13 (27.1)	12 (25)
Larger than 1 DD	20 (47.6)	12 (25)	11 (22.9)
Total	42	25	23

OD: right eye; OS: left eye; DD: disc diameter.

The size of the lesion showed a relationship with increasing age (p = 0.047; the median age of subjects with lesions larger than 1 DD: 55 years; the median age of those with lesions less than or equal to 1DD: 38.5 years—Statistical analyses were performed by Wilcoxon rank-sum test (Mann Whitney). Table 2 describes the clinical characteristics of ocular toxoplasmosis lesions. Subjects belonging to the low social stratum were more likely to present ocular toxoplasmosis than those belonging to the grouped higher social stratum (OR: 2.89; CI 1.2 to 6.97; p = 0.018). (Table 3). In eight eyes with OT (19%), presenting VA was < 20/50 (being two of them with presenting VA ≤ 20/200). There was one subject with bilateral OT and VA < 20/50 bilaterally; no subjects had OT bilateral VA ≤ 20/200. There was no significant difference in presenting VA in eyes with or without OT (OD p = 0.66; OS p = 0.87) and between subjects with or without OT, either considering the better-seeing (VA ≥ 20/40; p = 0.54) and the worse-seeing eye (VA ≤ 20/200; p = 0.41; Table 4). Statistical analyses were performed by the T-test.

**Table 3:** Results of binary logistic regression between study variables and ocular toxoplasmosis (n = 721 subjects).

Variable	OR	CI	p value
**Grouped social stratum**			
A2 + B1 + B2	Ref		
C1 + C2 + D + E	2.89	1.20–6.97	0.018*
**Sex**			
Male	Ref		
Female	1.37	0.70–2.70	0.36
**Age (years)**			
18–24	Ref		
25–34	1.60	0.19–13.42	0.68
35–44	1.04	0.12–8.92	0.97
45–54	0.06	0.07–5.34	0.66
55–64	0.84	0.10–7.12	0.87
> 64	0.67	0.08–5.75	0.71
**Educational level (years of study)**			
< 4	3.76	0.48–29.70	0.21
5–7	5.36	0.70–41.22	0.10
8–10	3.71	0.40–34.10	0.25
11–15	4.06	0.51–32.38	0.19
> 15	Ref		
**Consumption of undercooked meat**			
Yes	1.38	0.56–3.40	0.48
No	Ref		
**Consumption of raw vegetables**			
Yes	1.02	0.24–4.41	0.98
No	Ref		
**Felines at home**			
Yes	Ref		
No	1.33	0.69–2.55	0.39
**Contact with soil/garden**			
Yes	1.34	0.70–2.56	0.38
No	Ref		

Social categories classified according to the Brazilian Association of Research Companies (ABEP)^21^, being high (A1, A2, B1, B2) and low (C1, C2, D, and E) social stratum; (*) and bold, statistically relevant. Social stratum "E" composed of only two subjects, one with eye damage and the other without lesion.

Ref.: group used as a reference; CI: Confidence interval; OR: odds ratio.

**Table 4:** Presenting visual acuity between subjects and eyes with and without ocular toxoplasmosis (n = 718 subjects and 1436 eyes).

Visual acuity	≥ 20/40	20/50 to 20/160	≤ 20/200
Eyes with OT (n = 48)	34	12	2
Subjects with OT; better-seeing eye VA (n = 42)	38	4	0
Subjects with OT; worse-seeing eye VA (n = 42)	24	16	2
Eyes without OT (n = 1388)	1029	323	36
Subjects without OT; better-seeing eye VA (n = 676)	556	115	5
Subjects without OT; worse-seeing eye VA (n = 676)	446	201	29

Three subjects (six eyes), all without presumed toxoplasmosis lesions, were unable to report VA.

OT: ocular toxoplasmosis; VA: visual acuity.

## Discussion

Ocular toxoplasmosis was found in 5.8% of the subjects (6.7% in those with positive *T. gondii* serology). Most subjects with OT presented unilateral and peripheral lesions, and no active lesions were found. The presence of ocular lesions was associated with lower social stratum, and no association between the presence of OT in eyes and subjects and visual impairment was found.

Lower social stratum was identified as a risk factor for ocular toxoplasmosis, a similar result obtained in the city of Natal, Northeast Brazil^11^. It can be explained by the fact that lower social stratum subjects are more exposed to risk factors for contamination with the parasite due to less access to sanitary conditions, such as filtered water, for example^19,20^. In the present study, older age showed no association with the presence of ocular lesions, unlike other studies that reported this variable as a risk factor for ocular toxoplasmosis^15,16,21^. Larger lesions were most commonly found in older subjects, and this finding could be explained by their relative decreased immune response, which reduces the host’s ability to limit the parasite replication, leading to a larger scar^8^. Another potential explanation is the occurrence of confluent lesions due to multiple reactivations over time, since recurrences occur often adjacent to a retinal OT scar^3,22^.

A study with a sample composed of students and university staff in Colombia found ocular toxoplasmic lesions in 6.0% of participants^17^. In the United States, where *T. gondii* seroprevalence is considered lower than in Latin America or Africa, Holland estimated that 2% of those infected have ocular manifestations of the disease^23^. In Central Cuba, a medical records analysis demonstrated an incidence of 26.2 per 100,000 person-years and a prevalence of 33.9 per 100,000 person-years^24^. A population-based study conducted in Gana (West Africa) found a prevalence of 2.6% of lesions attributed to OT (3.0% among those with positive *T. gondii* serology), although authors did not report visual outcomes^16^. In our study, we found 5.8% with the sample with OT, and 6.7% considering only those with positive *T. gondii* serology, in agreement with most Brazilian community-based studies^13,14^.

In agreement with other community-based studies, most eyes and subjects with OT in our study presented VA ≥ 20/40^17,18^. This finding can be explained by the fact that most lesions found were located in peripheral retina, whereas the presence of central lesions is a known risk-factor for poor prognosis^9,10^. The lack of significant correlation observed between the OT lesions, and visual impairment should not be interpreted as a reduced disease burden. Considering the high frequency of positive serology (63.4% in this municipality) and the frequency of OT found (6.7% of those with positive serology), we could speculate for a similar hypothetical population of 1,000,000 adults the following numbers: 637,000 with positive *T. gondii* serology, 42,679 of those with OT, and 10,158 of them with at least one eye presenting central retinal OT lesion, and consequently, a higher impact in quality of life^25^.

Since the diagnosis of OT relies mostly on the clinical aspects of retinal lesions in the presence of positive *T. gondii* serology, it is expected that uncertainties and disagreement exist between examiners. This fact was also pointed by Jabs et al., who found a poor agreement between experienced examiners (kappa = 0.23)^26^. In our study, we reached a better agreement (Kappa = 0.37; p < 0.001); what we speculate gives greater reliability to our diagnoses.

As strengths of the study, we can point to the large sample size, the use of multiple experienced examiners to evaluate retinal lesions, the description of visual acuity of eyes with and without OT, and a risk-factor questionnaire. As limitations, although we had a high percentage of the city adult population examined (33.9%), subjects were not randomly chosen, and numbers found may under or over represent the actual number of persons with OT in this municipality. The higher attendance of women to the local health unit and the increased difficulty locating men during working hours for blood sample collection can explain the female preponderance in this study^27^. Also, pediatric population was not included in this survey. And finally, the time-lapse between blood collection and ocular evaluation could bias our analysis. To minimize this, those identified with a suspected lesion and negative *T. gondii* serology were invited for another blood draw.

Our results indicate that in this adult population, OT is found in one out of 15 individuals with positive *T. gondii* serology, and more frequently in the lower social stratum. Although most subjects with OT present normal VA, unilateral blindness associated with OT was identified in a reduced number of individuals. Further studies are still needed to understand the real-life frequency of ocular lesions, visual outcomes, and risk factors for poor prognosis of OT and make it possible to implement public policies aimed at prophylaxis of contamination and manifestation of ocular disease by the parasite.

## Methods

Our group recently published a *T. gondii* survey conducted in Cássia dos Coqueiros, (Southeast Brazil), and found a 63.7% seroprevalence in the adult population^27^. The present cross-sectional study examines the presence and risk factors for OT in this population, and is a sub-study of a large epidemiological investigation of multiple infectious diseases in the local adult population, led by one of our co-authors (Dr. Afonso Passos). The municipality of Cássia dos Coqueiros was chosen for the study for multiple reasons. The Ribeirão Preto Medical School has a local health unit there since the 1950s, with a record of good acceptance from the local population participation in research projects. Also, over the years, there were multiple informal reports of local health care providers regarding the potential impact of toxoplasmosis in the city and whether the intrinsic characteristics of numerous small, family-owned farms could play a role in it. Subject recruitment, blood collection, and laboratory tests were previously described^27^. Briefly, all adults living in Cássia dos Coqueiros (estimated eligible population of 2126 subjects)^28^ who attended the only local medical center, in 2010, for any reason, were invited to participate in the study. In addition, all inhabitants living in both urban and rural areas were encouraged to participate during the routine household visits performed by the community health agents of the Family Health Program. Peripheral blood was collected, and laboratory tests were performed in 990 subjects (46.6% of the eligible individuals) for the detection of IgG and IgM antibodies against *T. gondii* by chemiluminescent microparticle immunoassay (CMIA) using ARCHITECT Toxo IgM and IgG kits (Abbot, Wiesbaden, Germany), as part of the “Human Serum Bank project of Cássia dos Coqueiros, São Paulo” (approval number 3915/2010)^27^.

Between 2011 and 2013, subjects responded to a survey for the detection of potential risk factors for toxoplasmosis, including age, occupation, educational level, social stratum, time of residence in the municipality of Cássia dos Coqueiros, presence of felines at home, frequency of contact with soil, and ingestion of undercooked meat. The social stratum was divided into eight categories (A1, A2, B1, B2, C1, C2, D, and E; being A1 the highest and E the lowest) according to the Brazilian Association of Research Companies (ABEP)^29^, but to facilitate the calculations and the understanding of the results, they were grouped into high (A1, A2, B1, B2) and low (C1, C2, D, and E) social stratum. Rural and urban areas were removed from the analysis due to the municipality´s mixed rural/urban characteristics.

All 990 subjects were invited for a comprehensive ophthalmological examination, conducted in eight expeditions during weekends between 2016 and 2017. The examination consisted of presenting visual acuity (VA) measurement using an Early Treatment Diabetic Retinopathy Study (ETDRS) chart placed four meters away from the subject, performed by trained medical students under the supervision of ophthalmologists, and dilated ophthalmoscopy using a Heine Omega 100 indirect binocular ophthalmoscope (Herrsching, Germany) and a 20-diopter lens Volk (Mentor, Ohio—USA) auxiliary lens performed by retina or uveitis specialists. Examiners were masked for serological results at the time of the examination. Informed consent was obtained from all the participants.

Examiners evaluated the posterior pole, medium, and extreme retina periphery in 360° searching for ocular lesions that could be classified as “potential ocular toxoplasmosis” using the following criteria: a single focus of retinitis with or without a ‘headlight in the fog appearance’ (active), or a retinochoroidal scar with a variable degree of hyperpigmentation (inactive)^8^. The lesions were measured in disc diameters (DD), and in eyes with multiple lesions, the size of the most extensive lesion was registered. In bilateral cases, the most extensive lesion was taken into account, regardless of the eye. Regarding the retinal location, lesions were classified in either “central”, located within the large temporal vascular arcades^10^ or “peripheral”.

All potential toxoplasmic lesions were recorded as WMV files with a Keeler Vantage Plus LED indirect binocular ophthalmoscope (Malvern, Pennsylvania, USA) attached to a computer. WMV files were later analyzed by masked and experienced graders, who classified the lesions as “toxoplasmic,” “non-toxoplasmic” or “impossible to evaluate” (when images were not clear). Subjects were defined to have ocular toxoplasmosis if at least one suspected lesion was appointed by two masked graders, in the presence of positive anti-*T. gondii* IgG or IgM serology. A third (and more senior) grader analyzed the videos when there was a disagreement between the first two evaluators, and his opinion was considered the gold standard. Subjects with negative *T. gondii* serology who were classified as having “presumed ocular toxoplasmosis” were invited to repeat serological tests. Subjects who did not agree with the examination or did not respond after four invitations were excluded from the study. The study protocol was approved by the Committee on Ethics in Research of the Hospital das Clínicas da Faculdade de Medicina de Ribeirão Preto da Universidade de São Paulo (approval number 13.880/2014) and followed the Declaration of Helsinki.

### Statistical analysis

We used a “chi-square” test to assess the relationship between nominal variables. Among the quantitative ones, the difference in means was calculated by the “T-test” and the “Wilcoxon rank-sum test (Mann Whitney),” as most appropriate (parametric or not). Normality was checked with the Shapiro–Wilk test. We used the Odds Ratio (OR) value obtained through binary logistic regression to estimate the chances of association. The agreement between the examiners was evaluated by comparing their paired results using the Kappa calculation. The events were significant when they presented p < 0.05. We analyze the data using the Stata/MP 14.0 program (Lakeway, Texas—United States).

## Data Availability

All data are available upon request.
